# G-CSF and G-CSFR Modulate CD4 and CD8 T Cell Responses to Promote Colon Tumor Growth and Are Potential Therapeutic Targets

**DOI:** 10.3389/fimmu.2020.01885

**Published:** 2020-09-15

**Authors:** Ioannis Karagiannidis, Stephanie J. Jerman, Damian Jacenik, Brandon B. Phinney, Ruoxin Yao, Eric R. Prossnitz, Ellen J. Beswick

**Affiliations:** ^1^Division of Gastroenterology, Department of Internal Medicine, University of Utah, Salt Lake City, UT, United States; ^2^Department of Molecular Genetics and Microbiology, University of New Mexico Health Sciences Center Albuquerque, Albuquerque, NM, United States; ^3^Division of Molecular Medicine, Department of Internal Medicine, University of New Mexico Health Sciences Center, Albuquerque, NM, United States; ^4^Department of Cytobiochemistry, Faculty of Biology and Environmental Protection, University of Lodz, Lodz, Poland

**Keywords:** colorectal cancer, G-CSF, cytokines, G-CSF receptor, T cells, tumor microenvironment

## Abstract

Cytokines are known to shape the tumor microenvironment and although progress has been made in understanding their role in carcinogenesis, much remains to learn regarding their role in tumor growth and progression. We have identified granulocyte colony-stimulating factor (G-CSF) as one such cytokine, showing that G-CSF is linked with metastasis in human gastrointestinal tumors and neutralizing G-CSF in a mouse model of colitis-associated cancer is protective. Here, we set out to identify the role of G-CSF and its receptor, G-CSFR, in CD4^+^ and CD8^+^ T cell responses in the tumor microenvironment. MC38 colon cancer cells were injected into WT, G-CSFR^−/−^ mice, or Rag2^−/−^ mice. Flow cytometry, Real Time PCR and Multiplex cytokine array analysis were used for *in vitro* T cell phenotype analysis. Adoptive transfer of WT or G-CSFR^−/−^ CD4^+^ of CD8^+^ T cells were performed. Mouse tumor size, cytokine expression, T cell phenotype, and cytotoxic activity were analyzed. We established that in G-CSFR^−/−^ mice, tumor growth of MC38 colon cancer cells is significantly decreased. T cell phenotype and cytokine production were also altered, as both *in vitro* and *in vivo* approaches revealed that the G-CSF/G-CSFR stimulate IL-10-producing, FoxP3-expressing CD4^+^ and CD8^+^ T cells, whereas G-CSFR^−/−^ T cells exhibit increased IFNγ and IL-17A production, leading to increased cytotoxic activity in the tumor microenvironment. Furthermore, peritumoral injection of recombinant IFNγ or IL-17A inhibited colon and pancreas tumor growth compared to controls. Taken together, our data reveal an unknown mechanism by which G-CSF, through its receptor G-CSFR, promotes an inhibitory Treg phenotype that limits tumor immune responses and furthermore suggest that targeting this cytokine/receptor axis could represent a novel therapeutic approach for gastrointestinal, and likely other tumors with high expression of these factors.

## Introduction

Colorectal cancer (CRC) is the third most common human malignancy worldwide, and is a leading cause of cancer deaths in the United States (1, 2). While newer chemotherapy regimens have improved survival for CRC patients, immunotherapy has not been highly effective, limiting new therapy approaches. Understanding the tumor microenvironment and its role in immune regulation may be key steps to developing more effective therapies.

A critical component of the tumor microenvironment that drives immune cell responses is the cytokine/chemokine milieu. We have identified granulocyte colony-stimulating factor (G-CSF, also known as CSF3) as a tumor promoting cytokine that is produced by epithelial cells, monocytes/macrophages, fibroblasts and bone marrow stromal cells (3, 4). G-CSF is best known for its function in signaling to promote the survival, proliferation, differentiation and function of neutrophil precursors *via* interactions with the G-CSF receptor (G-CSFR) found on neutrophils. In fact, increased expression of G-CSF and its receptor is associated with various human malignancies, including lung (5), brain (6), breast, ovarian, bladder (7), gastric and colon cancers (8, 9). In particular, we have shown G-CSF and G-CSFR to be associated with metastasis in human gastric and colon cancer (10). Furthermore, tumors with high expression of G-CSF and G-CSFR are associated with increased tumor cell proliferation, migration and invasion as well as poor patient prognosis (10, 11). However, details of the mechanisms by which G-CSF/G-CSFR promote tumor progression and poor outcome remain elusive.

There are minimal studies suggesting G-CSF promotes immunosuppressive immune cell phenotypes. Previously, we demonstrated in a mouse model of colitis-associated cancer that mice treated with an anti-G-CSF antibody resulted in macrophages with decreased levels of pro-tumorigenic IL-10 and increased the expression of the anti-tumorigenic IL-12 (12). Additionally, one study showed that monocytes activated by G-CSF secrete IL-10 in a breast cancer model, which was enhanced in the presence of anti-CSF-1R antibody treatment (8). Although our group and later, this group have shown that macrophages activated by G-CSF promote tumor cell survival and progression, the effect of G-CSF on adaptive immunity and specifically the differentiation of other immune cells in the tumor microenvironment has not been examined.

The tumor microenvironment is comprised of different T cell populations that demonstrate either pro-tumorigenic or anti-tumorigenic activity. Thus, far, the most well-studied T cell subsets implicated in cancer immunity are the cytotoxic T lymphocytes (CD8^+^ T cells), T helper cells (Th1, Th2, and Th17) and regulatory T cells (Tregs) (13). In our previous study, we showed that G-CSF neutralization in the colitis-associated cancer model led to an increase in CD4^+^ and CD8^+^ T cells in mouse colons compared to isotype control treated mice (12). However, little information is available regarding the role of G-CSF in the regulation of T cell responses despite the fact that G-CSFR expression is universal in these cell types. Since our and other studies have begun to suggest that G-CSF may promote the induction/accumulation of IL-10-producing cells (12, 14, 15), we set out to determine whether G-CSF/G-CSFR specifically impacts CD4^+^ and CD8^+^ T cell responses.

In this study, we found that G-CSFR^−/−^ mice have significantly decreased tumor growth when injected with MC38 colon cancer cells. A decrease in IL-10 was detected, concurrent with an increase in IFNγ and IL-17A. Spleen-derived CD4^+^ T cells from G-CSFR^−/−^ mice also had decreased FoxP3 expression and IL-10 production along with increased expression of Tbet and IFNγ (indicative of a Th1 response) along with increased expression of RoRγ, and IL-17A (indicative of a Th17 response) compared to wild type (WT) CD4^+^ T cells *in vitro*. Adoptive transfer of G-CSFR^−/−^ CD4^+^ or CD8^+^ T cells into the peritumoral region of WT or Rag2^−/−^ mice led to significantly decreased tumor growth compared to T cells of WT mice. Cytokine changes and increases in cytotoxic activity, such as granzyme B and FAS expression, were evident, suggesting that G-CSF plays a role in both T helper and cytotoxic T cell activity in the tumor microenvironment. Furthermore, the changes in CD4^+^ and CD8^+^ T cell cytokines produced in the tumor microenvironment after G-CSFR^−/−^ cell injection led us to test the ability of IFNγ or IL-17A alone to inhibit tumor growth. Here, we found that administration of either IFNγ or IL-17A at biologically relevant concentrations was effective at reducing tumor growth, concurrent with supporting a cytotoxic immune response. In summary, we report here previously unrecognized pro-tumorigenic roles for G-CSF in gastrointestinal tumors through inhibiting CD4^+^ and CD8^+^ T cell responses by promoting IL-10 secretion and reducing cytotoxic responses. Overall, we suggest the previously unrecognized importance of G-CSF/G-CSFR regulation of T cell responses in shaping the tumor microenvironment and its potential as a therapeutic target in tumors with high expression.

## Materials and Methods

### Mice

C57BL/6 WT mice and B6(Cg)-*Rag2*^*tm1.1Cgn*^/J were obtained from the Jackson Laboratory. C57/BL/6 G-CSFR^−/−^ mice were obtained from Daniel Link at Washington University School of Medicine and backcrossed with WT mice. The animals were housed at constant temperature (22–24°C), relative humidity ~55% and maintained under 12 h light/dark cycle (lights turned on 8 a.m.) with access to standard chow pellets and tap water *ad libitum*. All animal studies were approved at the University of New Mexico Health Science Center IACUC or University of Utah IACUC.

### Cell Culture and Tumor Induction

MC38 cells were obtained from the NIH and subsequently Kerafast. PK5L1940 cells were obtained from Dr. Michael Gough at the Earle A. Chiles Research Institute (16). Cells were cultured in complete RPMI with 10% FBS, 1% penicillin/streptomycin, and 1% L-glutamine. Cells (2 × 10^6^) were injected in 100 μL of PBS (mixed 1:1 in Matrigel®) into the flank of 6–8 weeks old C57B/L6 and B6(Cg)-*Rag2*^*tm1.1Cgn*^/J mice. Both male and female mouse groups were used in multiple experiments. Some mice injected with MC38 cells were injected intratumorally with CD4^+^ or CD8^+^ T cells obtained from WT and G-CSFR^−/−^ mice. Treatments with 1 × 10^6^ T cells were administered at Day 1 and Day 7 after tumor cell injection. Recombinant mouse IL-17A and IFNγ (100 ng, Shenandoah Biotechnology) was injected three times per week for up to 4 weeks. Tumors were measured using calipers throughout the experiment and mice were euthanized between days 15 and 27 after tumor cell injection.

### T Cell Isolation and Culture

CD4^+^ and CD8^+^ T cells from WT and G-CSFR^−/−^ mouse spleens were isolated using the EasySep™ Cell Isolation & Cell Separation Kit (Stem Cell Technologies). Cells were cultured in complete RPMI with 10% FBS, penicillin/streptomycin, and L-glutamine. Cells were activated using T cell activation beads (ThermoFisher Scientific) coupled with anti-CD3 and anti-CD28 antibodies for *in vitro* assays. After 24 or 48 h in culture, cells were spun down at 300 × g for 5 min. Culture supernatants were collected (and stored at −80°C) for multiplex Luminex cytokine analysis (see below). The cell pellets were stored in RiboZol (VWR) for RNA extraction for qPCR or stained for flow cytometry. For injections into mice, freshly isolated cells were used without pre-activation.

### Flow Cytometry

T cell activation beads were removed and cells were washed with PBS containing 1% FBS and 2 mM EDTA. For evaluation of isolated CD4^+^ and CD8^+^ T cells, cells were blocked using normal rat serum for 15 min at room temperature and stained with anti-CD3-FITC (clone OKT3; eBioscience) and anti-CD4-PE (clone GK1.5; eBioscience) or anti-CD8-PE/Cy5 (clone 53–6.7; Biolegend) for 1 h to assess purity. Cells were fixed and permeabilized using the FoxP3 fixation and permeabilization kit from eBioscience according to the manufacturer's instructions. Subsequently, cells were incubated with the following anti-mouse antibodies in different combinations: FoxP3 (clone FJK-16s), Tbet (clone 4B10), GATA3 (clone TWAJ), RORγ (clone AFKJS-9), or isotype controls (all from eBioscience) for 1 h at room temperature. After washing, cells were analyzed by flow cytometry on a Guava EasyCyte Plus (Millipore) and analyzed using gauvaSoft Incyte software.

### RNA Isolation, Reverse Transcription, and Real Time PCR

T cell pellets were homogenized in RiboZol and RNA isolation was performed according to the manufacturer's protocol. RNA concentrations and purity were determined using a Nanodrop spectrophotometer (Thermo Fisher Scientific). cDNA synthesis was performed with qScript® cDNA SuperMix (Quantabio) in accordance with the manufacturer's protocol. Total RNA (100 ng/μL) was reversed transcribed using qScript® cDNA SuperMix reverse transcription mix (Quantabio) and the following PCR settings: 25°C for 5 min, 42°C for 30 min and 85°C for 5 min. Quantitation of mRNA was performed using the Real Time PCR method with FAM dye-labeled TaqMan® probes (Applied Biosystems). The reaction mixture consisted of cDNA, PerfeCTa® qPCR SuperMix (Quantabio), TaqMan® Assays and RNase-free water in total volume of 10 μL. The following validated TaqMan® Assays were used: FoxP3—Mm00475162_m1, IL-10—Mm01288386_m1, Gata3—Mm00484683_m1, IL-4—Mm00445259_m1, Tbet—Mm00450960_m1, IFNγ–Mm01168134_m1, RoRc—Mm01261022_m1, IL-17—Mm00439618_m1, Gzmb Mm00442837_m1, Prf1 Mm00812512_m1, Fasl Mm00438864_m1. Cycle parameters for TaqMan® Assays were as follows: initial denaturation at 95°C for 3 min, followed by 50 cycles of sequential incubations at 95°C for 15 s and at 60°C for 1 min. Results were normalized to the expression of Actb (β-actin). All experiments were performed at least as duplicates. Real Time PCR was performed on Applied Biosystem's StepOnePlus instrument (ThermoFisher Scientific). The endpoint used in real-time PCR quantification, CT, was defined as the PCR cycle number that crossed the signal threshold. Quantification of gene expression was performed using the comparative CT method (Sequence Detector User Bulletin 2; Applied Biosystems) and reported as the fold change relative to the mRNA of the mouse housekeeping gene, β-actin.

### Multiplex Cytokine Arrays

Tumor tissue pieces were cut to 8 mg (±0.5 mg) and incubated for 16 h in complete RPMI medium containing 10% FBS. Supernatants were collected analyzed for cytokines or soluble cytotoxic factors by Luminex bead array (MilliporeSigma, Billerica, MA) according to the manufacturer's instructions.

### Tumor Killing Assay

MC38 tumor cells were plated and WT or G-CSFR^−/−^ CD8^+^ T cells added in a ratio of 2 T cells per tumor cell. Cells were incubated for up to 24 h and co-cultures stained with anti-CD8 PE-Cy7 (clone 53–6.7) and Annexin V (Alexa Fluor 488).

### Statistical Analysis

Power analyses were performed to determine the sample size of the experimental and control groups to ensure that any effect would be statistically detectable. An alpha of 0.05 was used, and the minimum acceptable power was 0.80. A minimum of 5 animals per group (to allow for experimental error) with multiple independent experiments *in vivo* was used. Results were presented as the mean ± standard error of mean (SEM). Differences between means were evaluated by one-way ANOVA in GraphPad Prism 5. Values of *p* < 0.05 were considered statistically significant.

## Results

### G-CSFR^–/–^ Mice Have Reduced Tumor Growth and Altered Cytokine Production

**Figure 1:**
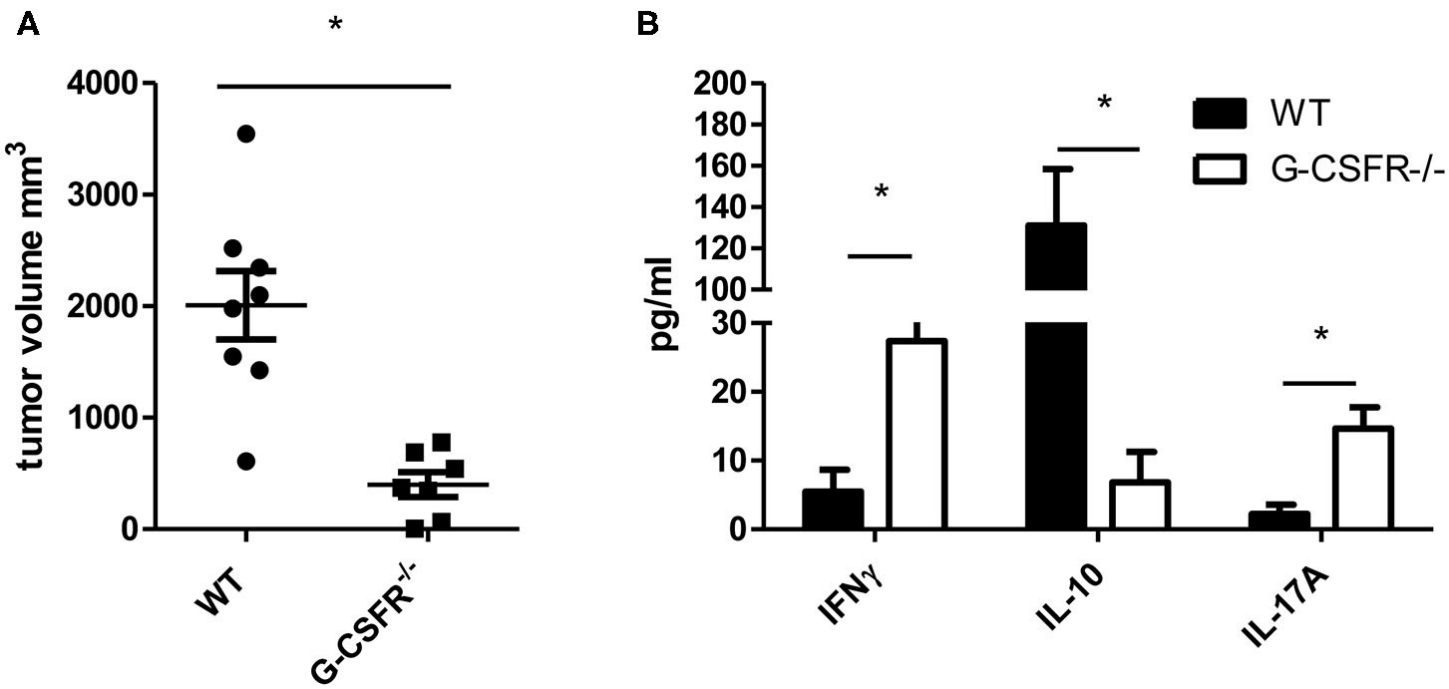
G-CSFR^−/−^ mice have **(A)** reduced tumor growth compared to WT mice and **(B)** changes in T cell-related cytokines in tumor. *N* =10, *p ≤ 0.05.

Since G-CSF has been shown to have pro-tumorigenic properties, we sought to examine how the tumor microenvironment and cytokine production are affected by G-CSF/G-CSFR. To address this, first we examined how tumors are impacted in G-CSFR^−/−^ mice compared to WT mice. Thus, WT and G-CSFR^−/−^ mice were injected subcutaneously with MC38 tumor cells and assessed for tumor growth. Fifteen days after tumor cell injection, the mice were sacrificed and tumors were extracted and measured. The G-CSFR^−/−^ mice showed a significantly reduced (4-fold) tumor burden compared to WT mice. Tumors in WT mice demonstrated a mean size of 2000 mm^3^ compared to 500 mm^3^ in GCSFR^−/−^ mice (Figure 1A), tumor curve in S1A. Next, the T cell-specific cytokine production was examined in both WT and GCSFR^−/−^ tumor tissues by multiplex array. Specifically, tumor tissue supernatants from WT and GCSFR^−/−^ mice tumors were assessed for the production of IFNγ, IL-10, IL-17A, and IL-4, which are considered general markers of Th1, Tregs, Th17, and Th2 cells, respectively. The analysis revealed a significant increase in the levels of IFNγ and IL-17A in G-CSFR^−/−^ mouse tumors when compared with the WT (Figure 1B). On the other hand, IL-10 levels were substantially decreased in G-CSFR^−/−^ derived tumors. These data suggest that G-CSF may be involved in regulating T cell responses in the tumor microenvironment.

### G-CSFR^–/–^ Activated CD4^+^ T Cells Show an Altered Phenotype Compared to WT CD4^+^ T Cells

**Figure 2:**
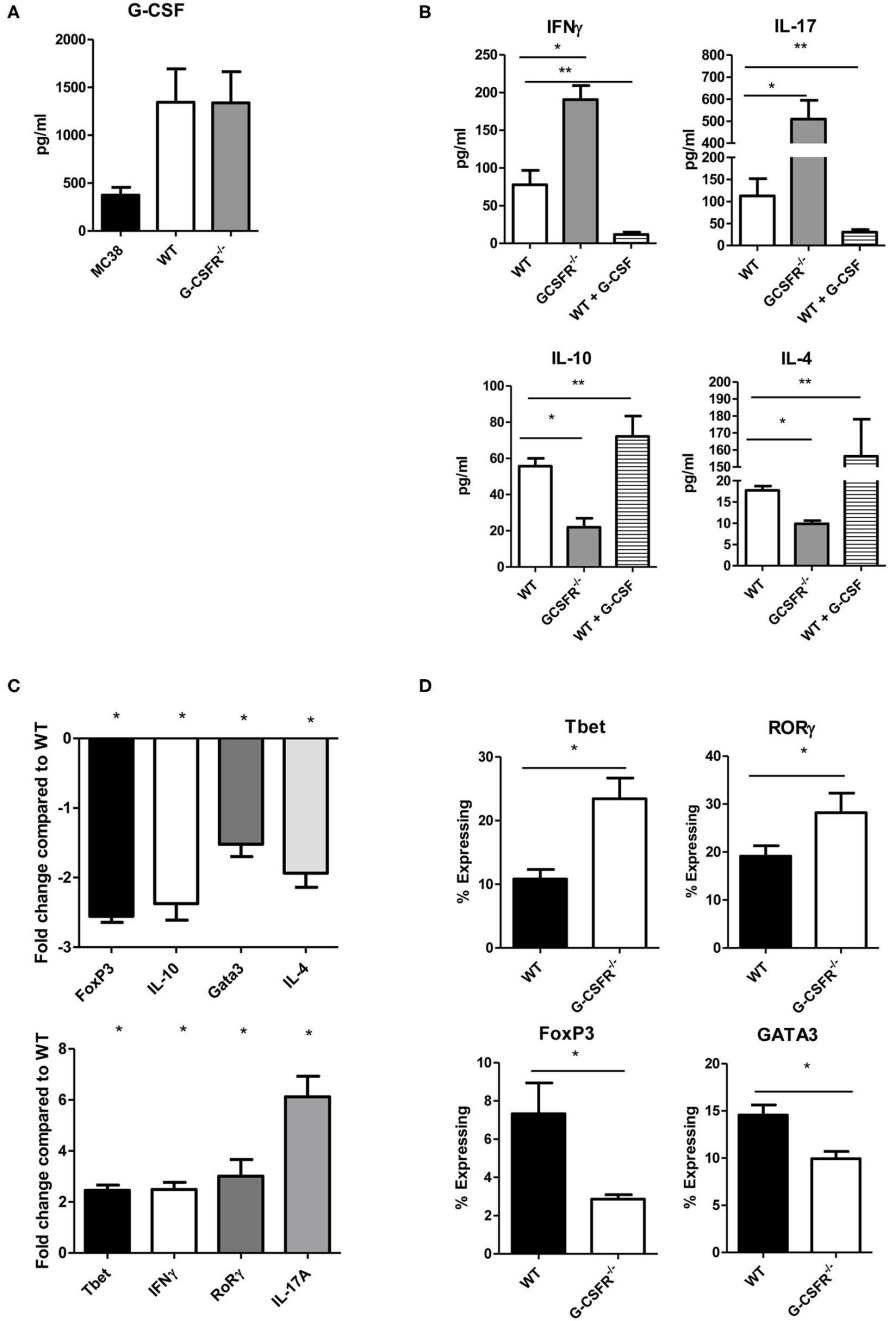
G-CSF/G-CSFR regulate CD4^+^ T cell phenotypes. In culture, **(A)** MC38 cells and both WT and G-CSFR^−/−^ activated mouse splenic CD4^+^ T cells produce G-CSF. **(B)** G-CSFR^−/−^ cells produce increased IFNγ and IL-17A and decreased IL-10 and IL-4 compared to WT cells. Addition of recombinant G-CSF to WT cells induces IL-10 and IL-4 and decreases IFNγ and IL-17A production. **(C)** A similar result is seen at the mRNA level where transcription factors and cytokines for T cell phenotypes are examined. **(D)** Also, transcription factor expression is altered by flow cytometry with G-CSFR^−/−^ cells showing increased Tbet and RoRγ staining and decreased FoxP3 and GATA3 staining. *N* = 6 for A-C and 9 for D. *p and **p ≤ 0.05.

Based on the results above that showed differences in secreted cytokines within the tumor between the WT and GCSFR^−/−^ mice, we next asked whether G-CSFR plays a role in the development of CD4^+^ T cell phenotypes. In order to examine this, CD4^+^ cells were isolated from spleens of WT and GCSFR^−/−^ mice, and stimulated by T cell activation beads *in vitro* for 24 h. Supernatants were collected and analyzed for cytokine production in the culture supernatant by multiplex array. First, we noted that G-CSF was produced by both MC38 tumor cell and CD4^+^ T cell cultures by both WT and G-CSFR^−/−^ T cells, suggesting the cytokine is present in the culture to act on the T cells (Figure 2A). As in the tumor tissues, IFNγ, IL-10, IL-17A, and IL-4 were measured and similar to tumor tissues, *in vitro* culture of T cells were found to have significantly increased levels of IFNγ and IL-17A, with G-CSFR^−/−^ CD4^+^ T cells, while IL-10 and IL-4 levels were decreased compared to WT (Figure 2B). Furthermore, when WT CD4^+^ cells were treated with 25 ng/ml of recombinant G-CSF (as titrated to be the adequate amount and previously published (10), IFNγ production was decreased below basal levels. Conversely, G-CSF treatment significantly decreased IL-17A production. G-CSF treatment of WT CD4^+^ T cells also increased IL-10 and IL-4 production. These findings suggest that GCSF directly affects CD4^+^ T cell phenotype and cytokine production.

In order to further investigate the role of G-CSF/G-CSFR in regulation of CD4^+^ T cell phenotypes, transcription factor and cytokine gene expression profiles were analyzed by qRT PCR with RNA extracted from cells from the cultures mentioned above. Similar to cytokine protein data, IFNγ and IL-17A expression at the mRNA level were significantly increased 2.5 and 6-fold, respectively in G-CSFR^−/−^ CD4^+^ T cells compared to CD4^+^ T cells from WT mice (Figure 2C). The transcription factors for Th1 (Tbet) and Th17 (RoRγ) were also increased 2.5 and 3.1-fold, respectively. Also in agreement with the data concerning cytokine production, IL-10 gene expression was decreased 2.4 fold and IL-4 2.0-fold in G-CSFR^−/−^ compared to WT CD4^+^ T cells. Also shown in Figure 2C, the transcription factors for Tregs (FoxP3) and Th2 (Gata3) were also decreased 2.6 and 1.5-fold, supporting the concept of a change in CD4^+^ T cell phenotype between WT and G-CSFR^−/−^ CD4^+^ T cells. Transcription factor analysis was further supported by flow cytometry of activated WT and G-CSFR^−/−^ CD4^+^ T cells for Tbet, FoxP3, RORγ, and GATA3. Similar to gene expression data, Tbet and RORγ expression were increased in the G-CSFR^−/−^ derived CD4^+^ T cells when compared with WT cells (Figure 2D), gating strategy in Figure S2. In contrast, GATA3 and FoxP3 expression levels were decreased in G-CSFR^−/−^ derived CD4^+^ T cells compared with WT CD4^+^ T cells. These results confirmed the findings of gene expression and cytokine production, suggesting that in the absence of G-CSFR, Th1 and Th17 phenotypes are favored, both *in vitro* as well as in the tumor microenvironment.

### Adoptive Transfer of G-CSFR^–/–^ CD4^+^ T Cells Reduces Tumor Growth in WT and Rag2^–/–^ Mice and Alters Cytokine Production in the Tumor Microenvironment

**Figure 3:**
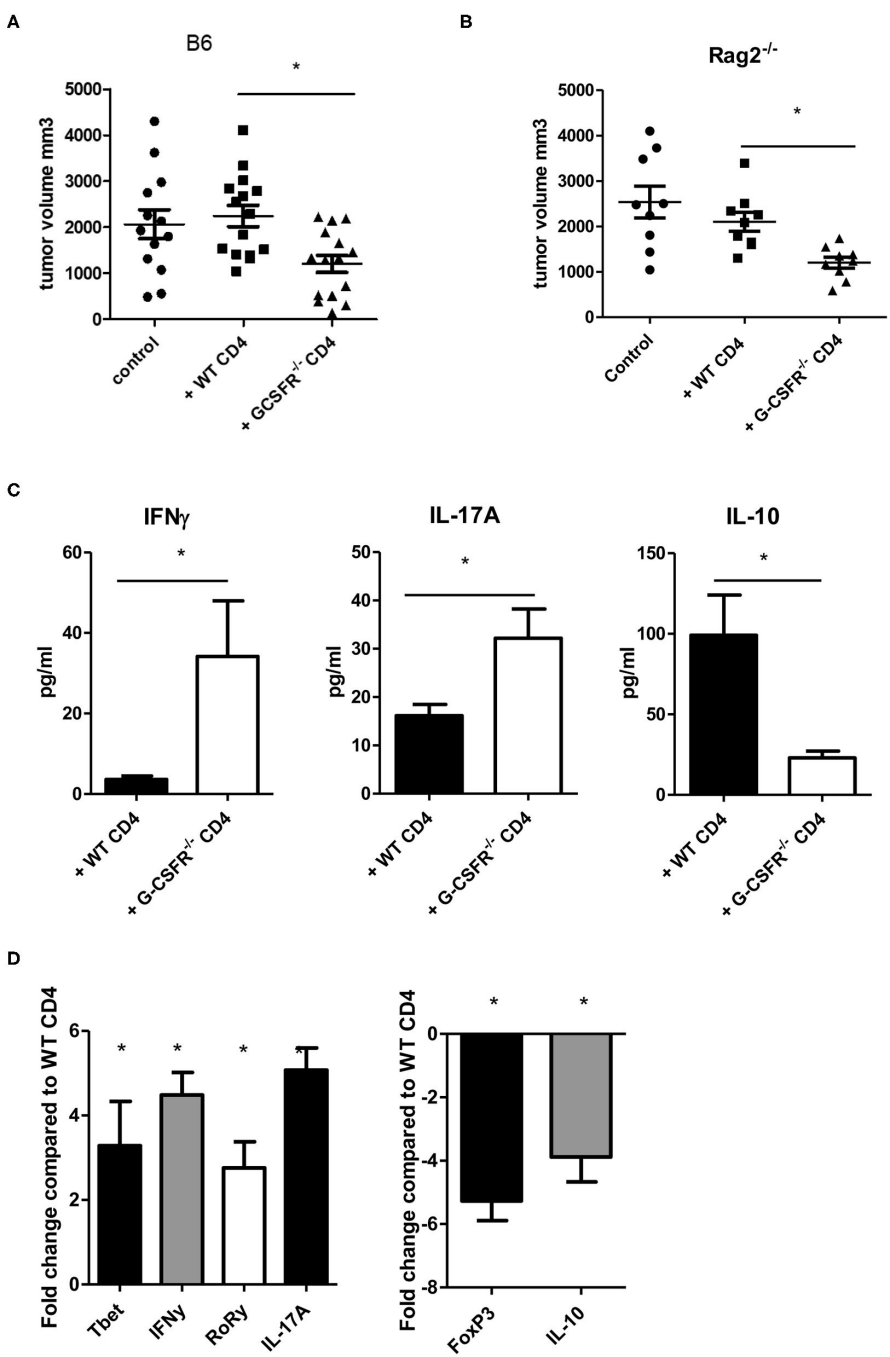
Adoptive transfer of G-CSFR^−/−^ CD4^+^ T cells reduces MC38 tumor growth in **(A)** WT and **(B)** Rag2^−/−^ mice accompanied by **(C)** increased IFNγ and IL-17A and decreased IL-10 in tumor tissue supernatants. **(D)** Gene expression showed similar changes in T cell-related transcription factors and cytokines. *N* = 8–10 **(A–C)** and 6 for **(D**). *p ≤ 0.05.

After exploring the role of G-CSF/G-CSFR *in vitro* in CD4^+^ T cell phenotypes, next, the impact of G-CSF/G-CSFR in CD4^+^ T cells in the tumor microenvironment on tumor growth was explored. To achieve this, WT and B6(Cg)-*Rag2*^*tm1.1Cgn*^/J mice were injected with MC38 colon tumor cells and adoptive transfer of CD4^+^ T cells isolated from WT or G-CSFR^−/−^ mouse spleens was performed. The B6(Cg)-*Rag2*^*tm1.1Cgn*^/J mouse strain has an inactivated RAG2 gene, therefore they are unable to initiate V(D)J rearrangement and fail to generate mature T and B lymphocytes (Rag2^−/−^). WT or G-CSFR^−/−^ CD4^+^ T cells (10^6^) were injected peritumoraly in WT and Rag2^−/−^ tumor bearing mice at Day 1, and Day 7. Mice were sacrificed between days 15 and 18 and the tumor volume was measured. Control tumors from both WT and Rag2^−/−^ mouse exhibited a similar pattern of growth, and tumors with WT CD4^+^ T cells injections did not differ significantly in size (Figures 3A,B). However, injection of G-CSFR^−/−^ CD4^+^ T cells resulted in tumors that were approximately half the size of control tumors and tumors injected with WT CD4^+^ T cells, growth curve in Figure S1B. Furthermore, in tumor tissue supernatants from Rag2^−/−^ mice, IFNγ and IL-17A were significantly increased, while IL-10 was significantly decreased (Figure 3C). IL-4 was not detectable in these tumors. Gene expression data further confirmed changes in CD4^+^ T cell phenotypes with Th1 and Th17 markers increased and Treg markers decreased (Figure 3D). However, overall the *in vivo* data are in agreement with the *in vitro* data showing a change in CD4^+^ T cell cytokine expression in mouse tumors along with a protective role with decreased tumor volume in the presence of G-CSFR^−/−^ T cells.

### Adoptive Transfer of CD8^+^ T Cells Into Rag2^–/–^ Mice Reduces Tumor Growth and Alters Cytokine Production in the Tumor Microenvironment

**Figure 4:**
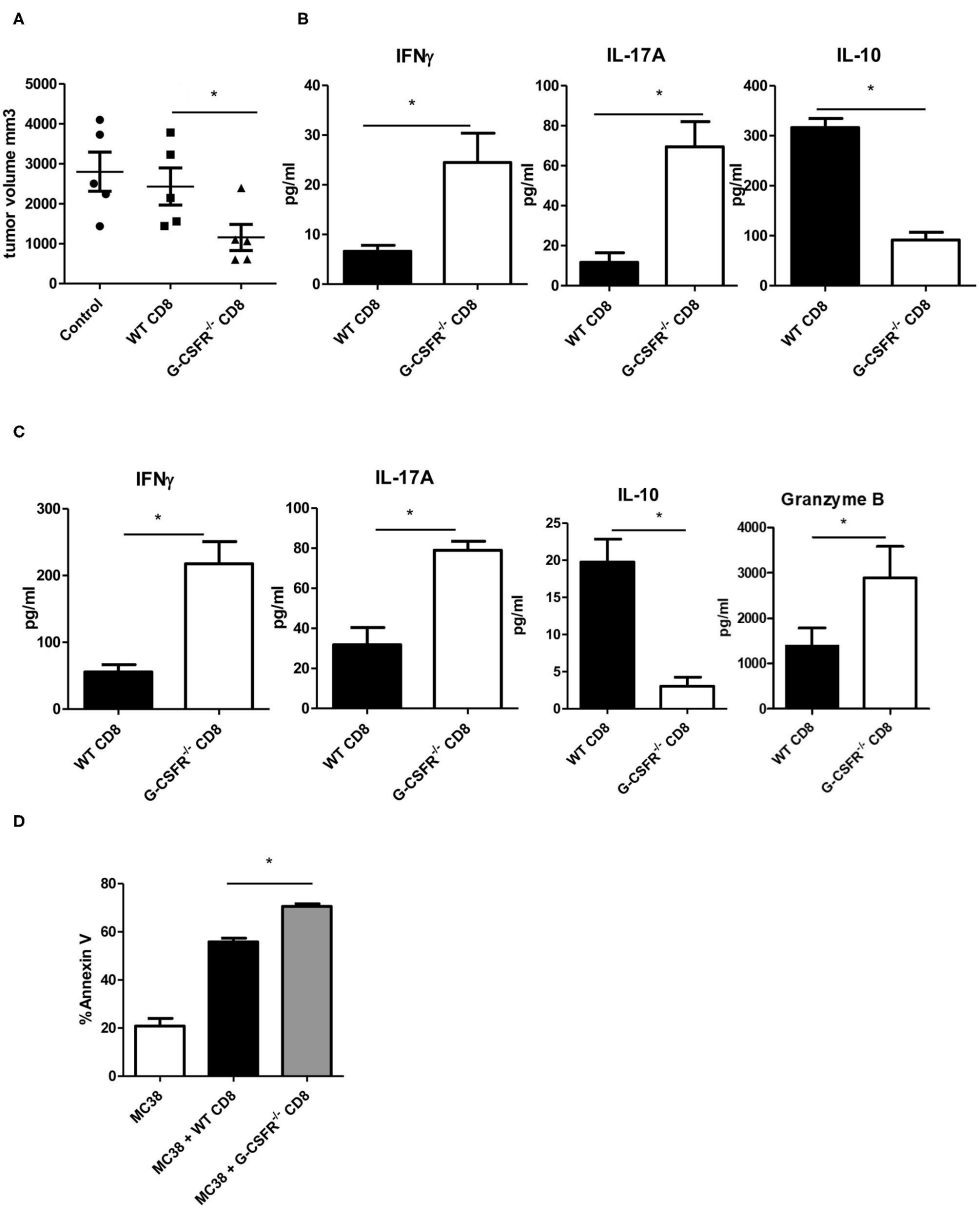
Adoptive transfer of CD8+ T cells inhibits MC38 tumor growth in **(A)** Rag2^−/−^ mice accompanied by **(B)** increased IFNγ and IL-17A and decreased IL-10 in tumor tissue supernatants and **(C)** changes in cytokines and granzyme B in cultured anti-CD3/anti-CD28 activated splenic derived CD8^+^ T cells. **(D)** *In vitro* tumor killing assay with MC38 cells and WT vs. G-CSFR^−/−^ CD8^+^ T cells. *N* = 5 for mouse studies and *N* = 6 for *in vitro* assays *p ≤ 0.05.

Because CD8^+^ T cells are also known to express cytokines that show similar phenotypes to CD4^+^ T cells (17), we also performed WT and G-CSFR^−/−^ CD8^+^ T cell adoptive transfer into Rag2^−/−^ mice with MC38 tumors. Since we did not see a significant difference between B6 and Rag2^−/−^ mice in Figure 3, only Rag2^−/−^ were used here. Similar to the CD4^+^ T cell study, control tumors and tumors injected with WT CD8^+^ T cells were not significantly different in size (Figure 4A). However, tumors injected with G-CSFR^−/−^ CD8^+^ T cells were approximately half the size of control tumors and those injected with WT CD8^+^ T cells, growth curve in Figure S1C. Furthermore, similar to the CD4^+^ T cell mouse group, tumors injected with G-CSFR^−/−^ CD8^+^ T cells showed a significant increase in IFNγ and IL-17A production, with a concomitant decrease in IL-10 production (Figure 4B). Since resulting tumors were decreased with adoptive transfer of CD8^+^ T cells, cytokine production was examined in *in vitro* assays in cells treated with anti-CD3/anti-CD28 activation beads. As seen in Figure 4C, activated CD8^+^ T cells show similar changes in cytokines as CD4^+^ T cells in Figure 2B. G-CSFR^−/−^ CD8^+^ T cells showed increased IFNγ and IL-17 and decreased IL-10 compared to WT CD8^+^ T cells. Furthermore, Granzyme B was also produced at higher levels in G-CSFR^−/−^ CD8^+^ T cells, suggesting that G-CSF plays a direct role in cytotoxicity. In an *in vitro* assay, CD8^+^ T cells isolated from mouse spleens were incubated with MC38 cells in a ratio of 2:1. Cells were stained with CD8 and Annexin V and CD8 gated out to analyze tumor cells. Annexin V staining was significantly increased in MC38 cells incubated with G-CSFR^−/−^ CD8^+^ T cells compared to cells incubated with WT CD8^+^ T cells (Figure 4D) with gating strategy in Figure S3. These data suggest that CD8 T cells have direct killing capacity of MC38 mouse tumor cells.

### Increased Cytotoxic Factors Are Present in Mouse Tumors Injected With GCSFR^–/–^ CD4^+^ or CD8^+^ T Cells

**Figure 5:**
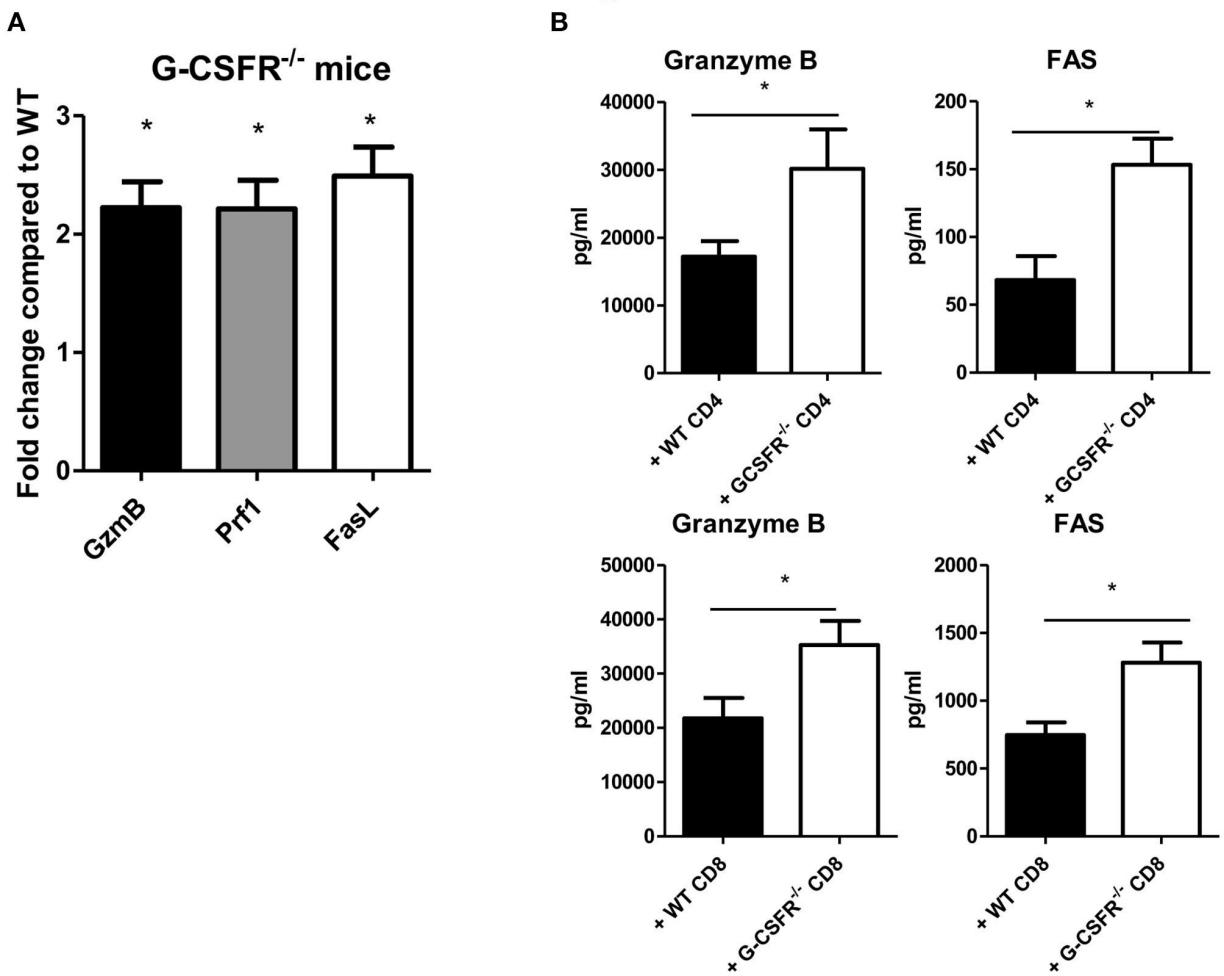
G-CSF/G-CSFR regulate cytotoxic factors where **(A)** G-CSFR^−/−^ mice show increased cytotoxic factor gene expression as do **(B)** tumor tissues from G-CSFR^−/−^ CD4^+^ and CD8^+^ adoptive transfer models in Rag2^−/−^ mice compared to WT. *N* = 6 for **(A,B)**, 10 for **(C)**, and 5 for **(D)**. *p ≤ 0.05.

To investigate possible mechanisms by which tumor growth was mitigated in G-CSFR^−/−^ mice and in the adoptive transfer models, cytotoxic factors were quantified in tumor supernatants and by gene expression analysis. Granzyme B and perforin were examined as cytotoxic factors while FAS and Fas ligand (FasL) were examined as tumor apoptotic markers. In the G-CSFR^−/−^ mice compared to WT mice, the expression of granzyme B, perforin, and FasL at the mRNA level was increased over 2-fold suggesting an increase in cytotoxic activity in the absence of G-CSFR in all immune cells, but not tumor cells (Figure 5A). Moreover, in both CD4^+^ and CD8^+^ T cell adoptive transfer models, granzyme B and FAS, which are available to detect as soluble factors, were detected with WT T cell groups, but significantly increased in groups receiving G-CSFR^−/−^ CD4^+^ or CD8^+^ T cells (Figure 5B). Notably, the overall level of FAS was 10-fold higher in the CD8^+^ T cell group compared to the CD4^+^ T cell group, suggesting the CD8^+^ T cells exert direct cytotoxic activity on tumors. However, since Rag2^−/−^ mice do not have CD8^+^ T cells, the increase of these factors in the CD4^+^ T cell group suggests that NK cells are also playing a role in the cytotoxic process.

### IFNγ and IL-17A Are Protective in MC38 and P5KL1940 Tumor Growth

**Figure 6:**
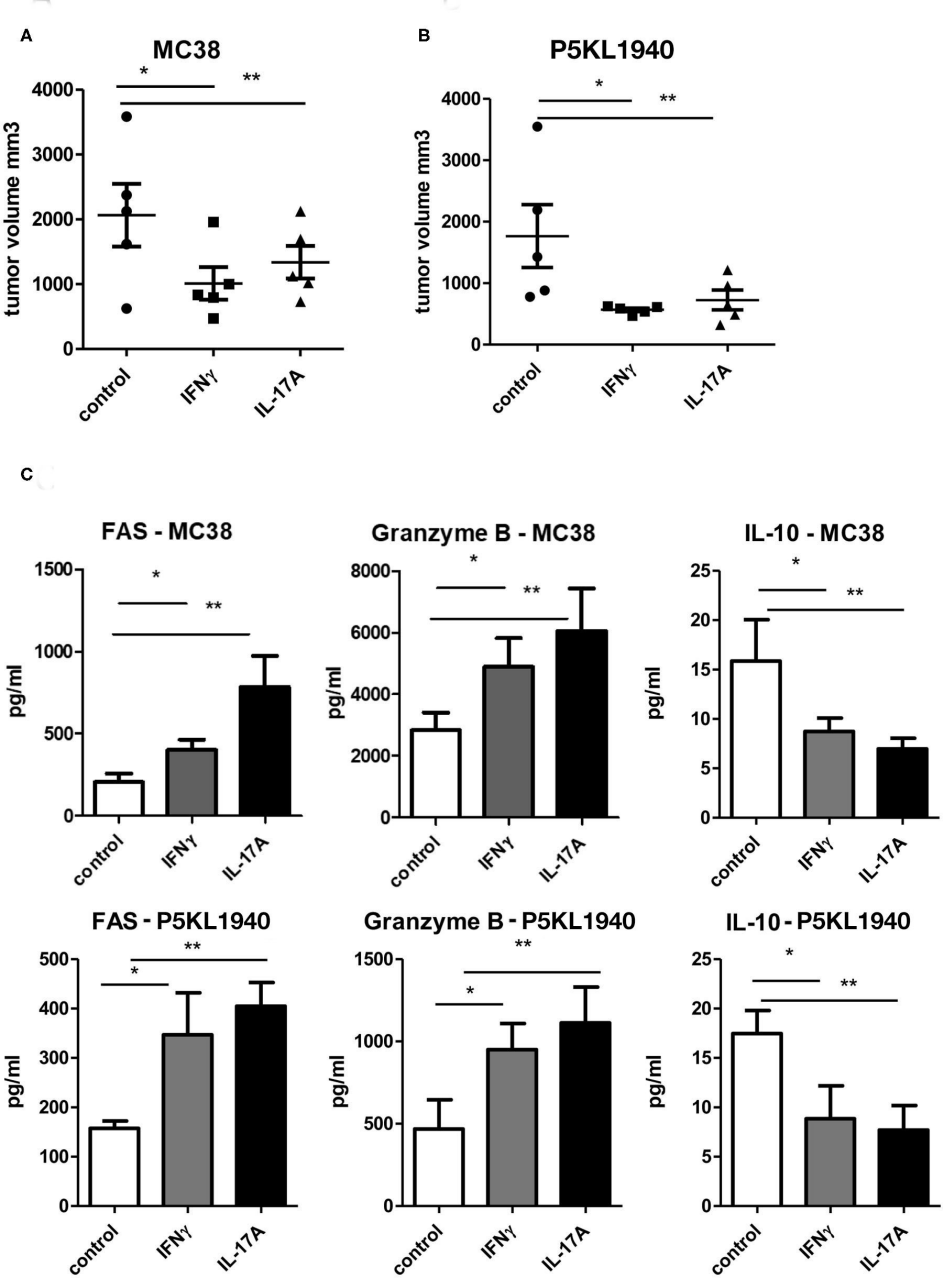
Recombinant IFNγ or IL-17A reduces **(A)** MC38 tumor growth, **(B)** PK5L1940 tumor growth and is accompanied by **(C)** increased tumor tissue FAS, Granzyme B and decreased IL-10. *N* = 5, *p and **p ≤ 0.05.

Since both CD4^+^ and CD8^+^ T cells from G-CSFR^−/−^ mice showed a similar effect when adoptively transferred into tumors, we also hypothesized that cytokines produced by CD4^+^ and CD8^+^ T cells could be playing a critical role in promoting tumor growth. Data above indicated that both IFNγ and IL-17A producing CD4^+^ and CD8^+^ T cells are increased upon G-CSFR^−/−^ T cell adoptive transfer. Therefore, to further examine the direct role of these cytokines in the tumor microenvironment, recombinant IFNγ or IL-17A were injected peritumoraly three times a week for up to 4 weeks. In these experiments, a second tumor model was examined, the pancreatic cancer line, PK5L1940. Similar results were seen with both tumor models in WT mice where either IFNγ or IL-17A significantly decreased tumor size (Figures 6A,B). The decreased tumor size was associated with increased cytotoxic factor granzyme B and the apoptosis marker FAS in tumor tissue supernatants (Figure 6C). Since IFNγ and IL-17A do not directly kill tumor cells (not shown), these data suggest that the increase in cytotoxic factors is responsible for tumor cell killing. Also, IL-10 was significantly decreased with IFNγ or IL-17A treatments, suggesting a decrease in inhibitory factors concomitant with an increase in cytotoxicity in two GI tumor models.

## Discussion

Granulocyte colony-stimulating factor is known as a regulator of hematopoietic growth, and is highly expressed upon infection and inflammation. Its most well-known function is the maturation and mobilization of polymorphonuclear neutrophils (PMNs). However, we found that it is highly expressed in both colitis-associated (12) and here tumor cell transfer models. We previously showed that both gastric and colon tumor cells express G-CSF and G-CSFR and can directly promote tumor cell proliferation. Furthermore, our previous findings suggest a strong association between both G-CSF and G-CSFR in colon and gastric cancer metastasis (10). In agreement with these findings, in human tumors there are various reports of extremely aggressive G-CSF producing tumors in multiple cancer types, such as lung cancer (18, 19), cervical cancer (20), meningioma (21), melanoma (22), breast cancer (8) and prostate cancer (23), demonstrating the important and modulatory effect of G-CSF in tumor progression and metastasis.

Little is known regarding the effects of G-CSF on lymphocytes. Some groups have suggested that CD4^+^ T cells in the presence of G-CSF experience suppressed CD28 signal transduction, which can inhibit their activation and survival. Moreover, it is possible that G-CSF-activated monocytes could impair CD4^+^ T cell function by the induction of apoptosis through the Fas-Fas ligand pathway (24, 25). Recently it was reported that upon G-CSF treatment in culture, CD8^+^ cells show decreased levels of activation proteins, suggesting that G-CSF directly affects, the functionality of CD8^+^ cells in a negative way (26). It was also reported, that in 260 patients treated with chemotherapy, G-CSF induced both acute and chronic graft-versus-host disease upon allogeneic hematopoietic stem-cell transplantation (27). In addition, treatment of peripheral blood cells with G-SCF can increase the production of γδ-Treg cells *in vivo* and *in vitro*, and support acute graft-versus-host disease in G-CSF-mobilized allogeneic peripheral blood stem cell transplantation (28). Furthermore, G-SCF promotes the mobilization of Tregs, by decreasing the activation of CXCR4 (29), while other literature suggests that G-CSF may support regulatory T cells (14, 30). Specifically, the addition of G-CSF in hematopoietic stem cell-derived Tregs, promotes their expansion and maintains their phenotype and cytokine production. Lastly, isolated CD4^+^ T cells treated *in vivo* with G-CSF acquire a Treg phenotype, with high expression of IL-10, and low expression of TGFβ, IL-2, and IL-4 (14). Since regulatory T cells are associated with a poor anti-tumor response (31), we sought to determine the role of G-CSF on T cells in the tumor microenvironment.

In this study, we observed that G-CSFR^−/−^ mice experienced significantly decreased tumor growth when injected with MC38 colon cancer cells. Since these are full knockout mice, the tumors expressed G-CSF and G-CSFR, while the immune cells in the mice were incapable of responding to G-CSF. The changes in tumor growth were associated with changes in T cell-associated cytokine production. IL-10 was substantially decreased in G-CSFR^−/−^ mice, which is in agreement with previous reports (26, 32), suggesting G-CSF supports regulatory T cells. Furthermore, a drastic increase in IFNγ and IL-17A was observed. Since IL-17A may also be produced by neutrophils, we did rule out a decrease in granulocytes in the tumor by testing for GSR expression, which was unchanged. Thus, these results point to a regulatory role for G-CSF on T cell phenotypes as the major producers of IFNγ and IL-17A besides granulocytes.

In *in vitro* studies, G-CSFR^−/−^ spleen-derived CD4^+^ T cells had decreased FoxP3, IL-10, GATA3, and IL-4 production along with in an increase in Tbet, IFNγ, RoRγ, and IL-17A production, compared to WT CD4^+^ T cells. It is well-established that IFNγ- and Il-17A-producing effector T helper cells, called Th1 and Th17 respectively, are dominant modulators/effectors that provide anti-tumor activity (13, 33). Although IFNγ-producing Th1 cells are accepted as having anti-tumorigenic function (34, 35), the role of Th17 cells is not completely understood. Some studies report that the IL-17A receptor promotes early colorectal tumorigenesis (36, 37), while others studies suggest that CRC-derived Th17 cells affect the aggregation of cytotoxic CD8^+^ T cells within the tumor (38, 39). Our results from the *in vitro* experiments showed that the absence G-CSFR promotes Th1 and Th17 phenotypes while Tregs and Th2 cells appear to be reduced. These results are in agreement with the pro-tumor promoting activity of Tregs in human CRC (40) and could also provide part of the explanation as to why tumor size was reduced in G-CSFR^−^/^−^ mice. More specifically, it has been reported that depletion of FoxP3^hi^ Treg cells from CRC tumors may promote antitumor immunity, while patients with gastrointestinal cancer present with high levels of Treg cells (41, 42). The role of Th2 cells in cancer remains controversial with studies supporting both anti- and pro-tumor activity. In mice with MHC class II-negative myeloma, treatment with Th2 cells led to complete clearance of antigen-producing tumor cells by inducing type II inflammation and recruiting M2 macrophages to the tumor site (43). On the other hand, the pro-tumor activity of Th2 cells in pancreatic cancer, where these cells seem to have an active role on tumor progression, may explain the poor prognosis and reduced survival in 69 surgically resected pancreatic tumor patients (44). However, in our experiments, as expected of B6 mice, a strong Th2 response was not present, demonstrated by the lack of IL-4 detection in adoptive transfer models.

Our adoptive cell transfer experiments sought to confirm that G-CSFR^−^/^−^ CD4^+^ cells, which were prevalent in anti-tumor phenotypes, could transfer those properties to the tumor microenvironment of Rag2^−/−^ mice, which lack T cells. The same experiments were also performed with adoptive transfer of the GCSFR^−^/^−^ CD8^+^ cells since CD8^+^ T cells have distinct phenotypes similar to CD4^+^ T cells (Tc1, Tc2, Tc17), and the differentiation of those CD8 subsets is determined by the presence of corresponding cytokines such as IFNγ, IL-4, and IL-17 (17). Transfer of GCSFR^−/−^ CD4^+^ and CD8^+^ cells yielded similar effects in reducing MC38 tumor growth when injected in Rag2^−/−^ mice. CD8^+^ T cells are present in different stages of CRC, with high levels of cytotoxic T cell infiltration suggesting an anti-tumor immunity (45). Intratumoral CD8^+^ cell infiltration may act to suppress the micrometastasis of cancer cells into other tissues (46). The density of CD8^+^ infiltration is decreased with tumor progression and can be used as a marker for better prognosis in patients with CRC (47, 48).

The reduction in tumor size was accompanied by changes in cytokines and increases in cytotoxic activity. Granzyme B and Fas were elevated in both G-CSFR^−^/^−^ CD4^+^ and CD8^+^ treated tumors, suggesting that G-CSF plays a role in both T helper and cytotoxic T cell activity in the tumor microenvironment. With CD4^+^ T cell adoptive transfer into Rag2^−/−^ mice, NK cells must play a role since CD8^+^ T cells are not present, yet a cytotoxic response was observed. These data are in line with our previous work where we found that, in the colitis-associated cancer model, NK cells were increased in the colons of mice treated with anti-G-CSF antibodies (12). Furthermore, a similar correlation between G-CSF and NK cells was present in two additional studies where G-CSF-mobilized hematopoietic stem cells from 32 donors resulted in a downregulation of NK cells (49). In another study, a five day treatment of hematopoietic stem cells with G-CSF led to comparable results with a downregulation of NK cell number (50).

The changes in T cell cytokines produced in the tumor microenvironment following G-CSFR^−/−^ cell injection led us to test the ability of IFNγ or IL-17A alone to inhibit tumor growth. Although IFNγ is known to support anti-tumor immunity, we further showed that it is sufficient to shrink tumors in both colon and pancreas. Importantly, conflicting results exist regarding the role of IL-17A in CRC, with both pro- or anti-tumorigenic activity observed (38). Since IL-17A has not been directly administered in biologically relevant concentrations in GI tumors, we performed this experiment and found it was effective in reducing tumor growth, concurrent with supporting a cytotoxic immune response. Thus, these data suggest a potential role of IL-17A as a therapeutic factor.

In conclusion, we report previously unrecognized pro-tumorigenic roles for G-CSF in gastrointestinal tumors through inhibition of CD4^+^ and CD8^+^ T cell responses by promoting IL-10 and reducing cytotoxic responses in IFNγ- and IL-17A- dependent mechanisms. We conclude that the G-CSF/G-CSFR-mediated regulation of T cell responses plays a critical role in shaping the tumor microenvironment by inhibiting anti-tumorigenic immune responses in at least two GI tumor types. Tumors with high expression of G-CSF could be considered as resistant to immunotherapy. Particularly novel is the previously unreported role we found for G-CSF/G-CSR blockade in promoting cytotoxic immune cell responses, which may be harnessed for improved therapy approaches. Furthermore, anti-G-CSFR therapy could be considered for some tumor types to combine with currently available immunotherapy approaches.

## Data Availability Statement

Data supporting the conclusion of this article is available by the authors.

## Ethics Statement

The animal studies were reviewed and approved by University of New Mexico Health Sciences Center and University of Utah Health IACUCs.

## Author Contributions

EB initiated the project. IK, SJ, BP, DJ, RY, and EB performed experiments. EB and EP supervised experiments. IK, SJ, BP, DJ, RY, EP, and EB analyzed data and participated in manuscript preparation. All authors contributed to the article and approved the submitted version.

## Conflict of Interest

The authors declare that the research was conducted in the absence of any commercial or financial relationships that could be construed as a potential conflict of interest.

## Abbreviations

CRC: colorectal cancer
G-CSF: granulocyte colony-stimulating factor
Regulatory T cells: Tregs
WT: wild type
FasL: FAS Ligand
PMNs: polymorphonuclear neutrophils.
