# Supplementary material for: G-CSF and G-CSFR Modulate CD4 and CD8 T Cell Responses to Promote Colon Tumor Growth and Are Potential Therapeutic Targets
Source: Front Immunol. 2020 Sep 15;11:1885. doi: 10.3389/fimmu.2020.01885 (PMC7522314; doi:10.3389/fimmu.2020.01885)
Supplement: Supplementary file 1 [file Data_Sheet_1.PDF]

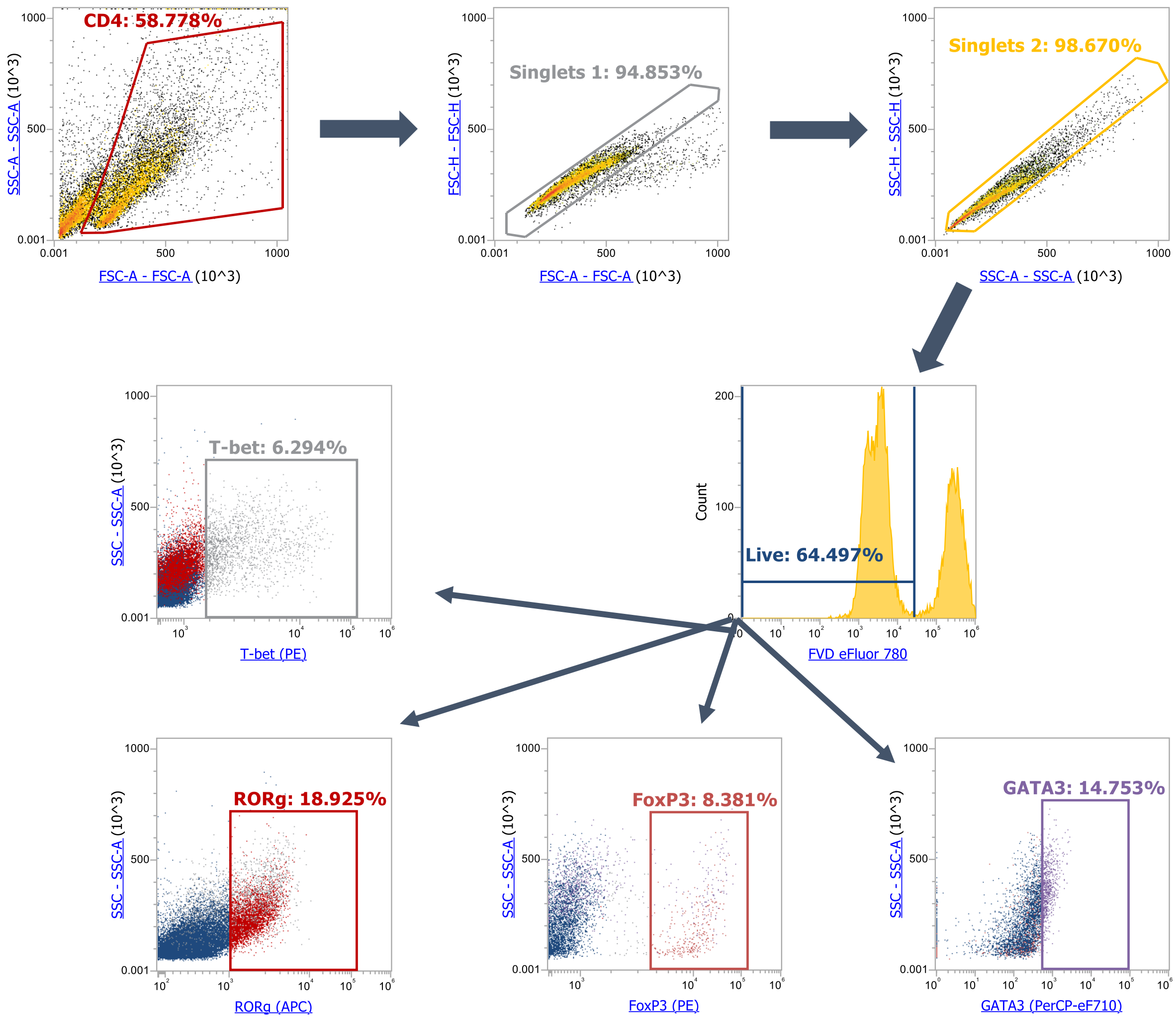

Figure S1. Gating Strategy for Transcription Factor staining in CD4+ cells isolated from mouse spleen. Cells were first gated for debris using forward and side scatter (FSC-A vs SSC-A). Next, doublets were excluded twice using forward scatter (FSC-A vs FCS-H) and side scatter (SSC-A vs SSC-H). Dead cells were excludes using Fixable Viability Dye e-fluor 780. Gating on live cells, positive populations for transcription factors, RORγ, FoxP3 and GATA3 were selected. Positive gates were determined using FMO, single color and isotype controls.

A

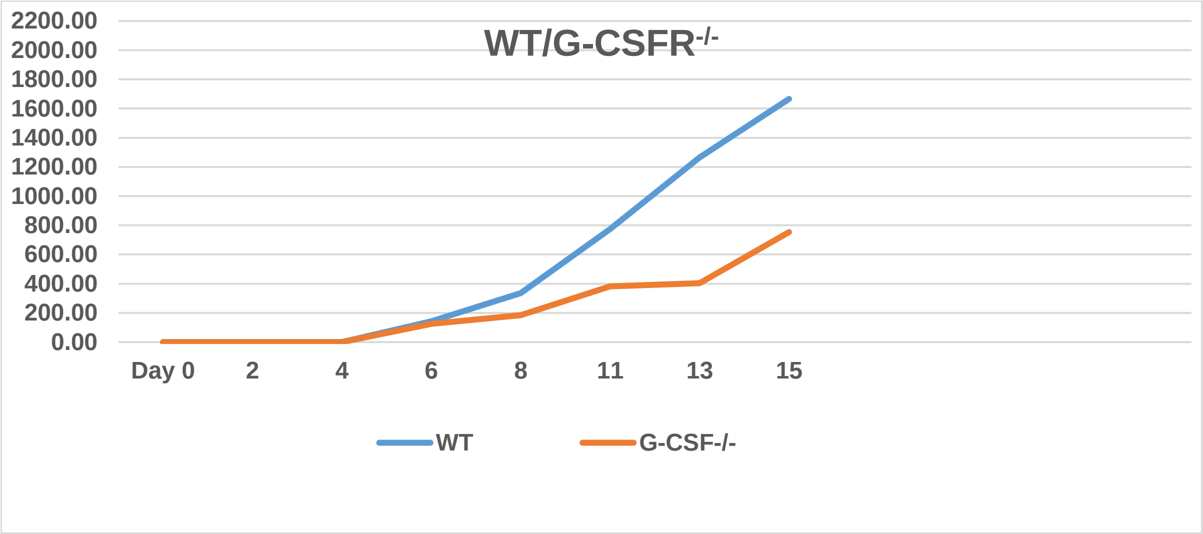

B

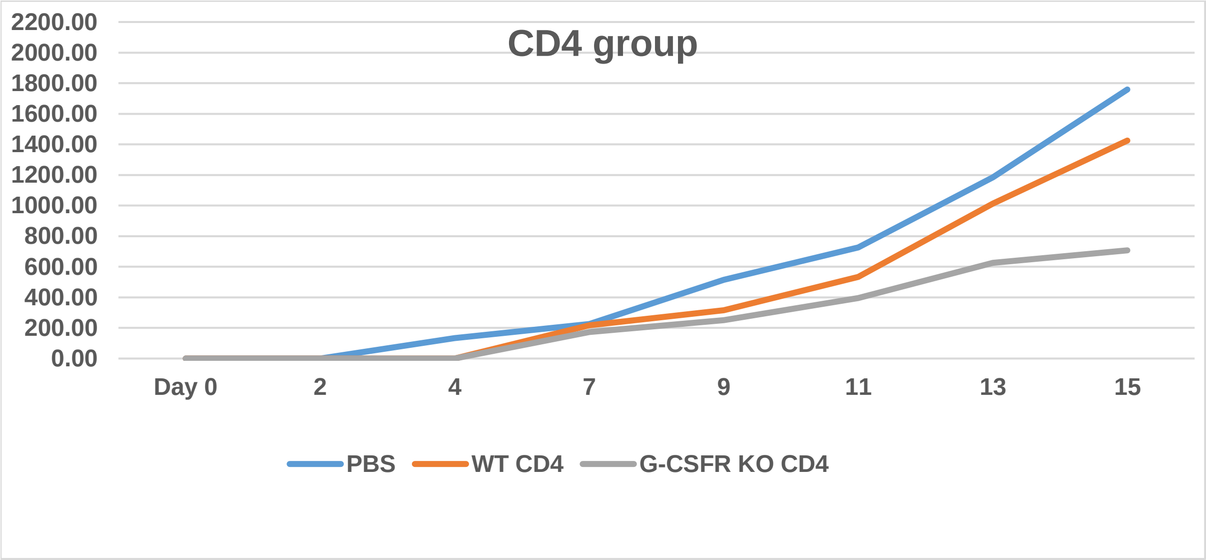

C

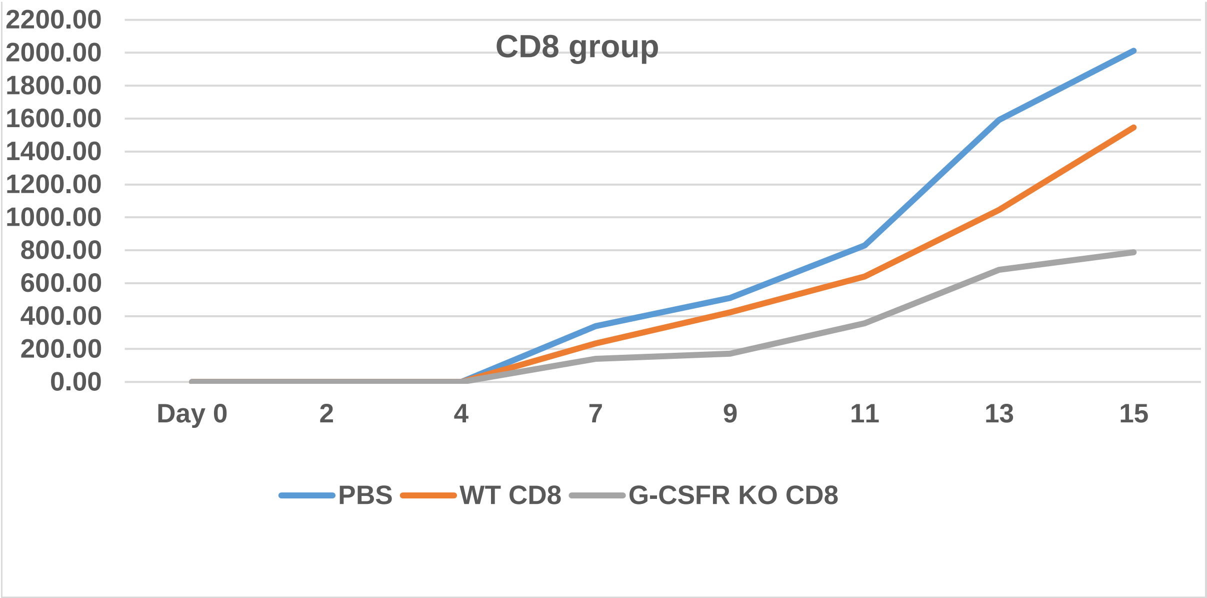

Figure S2. Growth curves of MC38 tumors in A) WT vs G-CSFR$^{-/-}$ mice, B) Rag 2$^{-/-}$ mice with CD4^{+} T cell adoptive transfer, and C) Rag 2$^{-/-}$ mice with CD8^{+} T cell adoptive transfer

Supplement:

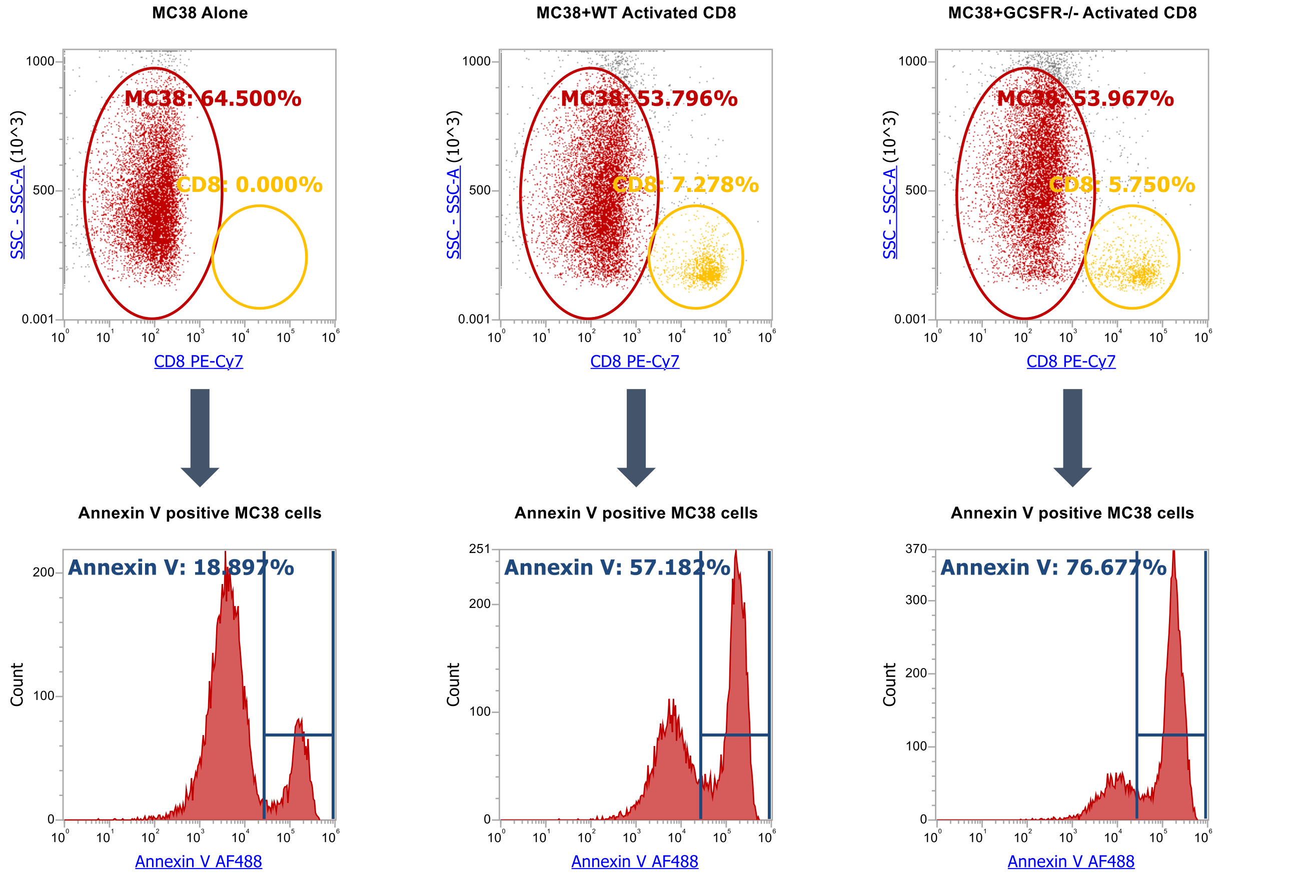

Figure S3. CD8 Tumor cell killing assay gating strategy. MC38 tumor cells incubated for 24 hours alone or with WT or G-CSFR$^{-/-}$ CD8^{+} T cells show changes in Annexin V staining.
